# The Dentists' Register

**Published:** 1923-01

**Authors:** Norman C. King

**Affiliations:** *Registrar*, Dental Board of the United Kingdom, 44 Hallam Street, London, W.1.


*To the Editor of* The Hospital and Health Review.

### The Dentists' Register.

*To the Editor of* The Hospital and Health Review.

Sir,—May I ask you to be good enough in your next issue to remind those persons who are liable to pay the fee for the retention of their names on the Dentists' Register, and have not yet done so, that, unless the remittance is received on or before December 31, their names will not appear in the Dentists' Register for 1923, and that, if they practise after December 31, they will render themselves liable to prosecution, and, on conviction, to a heavy fine.

Yours faithfully,
Norman C. King,
*Registrar,*
Dental Board of the United Kingdom,
44 Hallam Street, London, W.1.

Publicity is asked by the Dental Board of the United Kingdom for the two following points in connection with :

(*a*) A concession to dental students with regard to *time.* Dental students who are actually registered as pupils or apprentices of practitioners who are on the register may in certain cases obtain a concession which will have the effect of shortening the period, by not exceeding twelve months, of study required. This point, however, is subject to the requirements of the Licensing Body, whose qualification is sought, and inquiries should be made from the Body on the subject.

(*b*) A concession with regard to *fees* to be paid by those whose names have been added to the Dentists' Register by the Act.

There are three classes of applicants seeking to be excused from paying the fee, viz. : (1) Those who were prevented by war service from qualifying before July 28, 1921. (2) Those who passed the examination before the Act was passed, and failed through neglect to register before July 28, 1921. (3) Those who passed the examination before the Act was passed, but were prevented from registering owing to delay on the part of the Licensing Body, before July 28, 1921.

The Registration Committee of the Dental Board resolved that in (1) and (2) the applicants should be required to pay the fee for retention of their names on the Register, but that with regard to (3) if the applicant had passed the examination before July 28, 1921, and it could be shown he was prevented from registration by delay on the part of the Licensing Body, the annual fee should not be required.

### POST GRADUATE COURSE ON INFANT AND CHILD WELFARE.

A course of twelve lectures to qualified practitioners on the management and feeding of infants and young children will be given by Dr. Eric Pritchard (Medical Director of the Hospital) at the Infants' Hospital, Vincent Square, S.W., on Mondays and Tuesdays at 6 p.m., from January 15th to February 22nd inclusive.

Students taking this course will be entitled to attend the round-table consultations held by Dr. Pritchard on Tuesdays and Fridays at 2 p.m., with cases in the wards of the Infants' Hospital. Opportunities will also be afforded to students of visiting the Nursery Training School, 3 Wellgarth Road, Golder's Green on Saturday afternoons at 3 p.m.

Tickets (£2 2s. for the course) and further information can be obtained from the Secretary, The Infants' Hospital, Vincent Square, S.W.1.

### THE ASSOCIATION OF CERTIFIED BLIND MASSEURS.

The work of this association, which is to train students in massage, remedial gymnastics and medical electricity, is indeed of far-reaching importance, since at the present time there are members resident in Australia, New Zealand, Canada and South Africa. During the two years' training the students receive practical instruction in the massage departments of various London hospitals, whilst theoretical instruction is given at a training school, where in addition to raised diagrams, there are provided a number of works on massage written in embossed type. The members of the association are for the most part soldiers blinded in the war, and, as they all have to pass the same examination as sighted candidates, their competence may be thoroughly relied upon.

The National Institute for the Blind, whose address is 224-8 Great Portland Street, W.1, will, I am sure, be pleased to give further information to any inquirer.

### A TRAGIC PICTURE.

In a communiqué issued by the secretariat of Dr. Nansen for the relief of Russia the following details are given of the present position of the doctors and health officers in the province of Nikolaev, in the Ukraine :—

The assistance given to doctors has, so far, been negligible, and no organisation for their relief exists in the province. A few doctors who have friends abroad have occasionally received parcels of foodstuffs. All goes to show that in the coming winter the famine and conditions of life generally will be still worse than in the past, unless help is sent from abroad. The doctors receive their salaries only after long delays. Their tragic position continues to grow worse. During the past winter some supplies were officially distributed to them, but even this relief has now come to an end. The medical staff must meet their necessities as best they can. Very many doctors are only able to subsist by the sale of their furniture, or any objects which they may possess. Even those who have the best practices are in need of clothing. The poorer doctors who live in remote districts have to rely on the help of such peasants who have themselves managed to escape ruin.

The situation in the province of Nikolaev gives some idea of the conditions which exist in other parts of the country. Without the official ration, which consists of an English pound of maize distributed to a portion of the staff in the hospitals, life would have been impossible in these establishments. This relief has now come to an end. At the present moment a hospital doctor, if he is paid, receives 27 million roubles (in September this was equivalent to £1 but at the present moment it is less than 8s.). The hospital sisters receive about 20 million roubles, and other members of the staff still less. A great number of persons belonging to the staff of the hospitals have died at their posts.

This picture clearly shows the tragic position of the Russian doctors in the famine districts, and the urgent necessity for providing them with relief.

